# Targeted Genome Reduction of *Pseudomonas aeruginosa* Strain PAO1 Led to the Development of Hypovirulent and Hypersusceptible rDNA Hosts

**DOI:** 10.3389/fbioe.2021.640450

**Published:** 2021-03-11

**Authors:** Mélanie Grosjean, Sophie Guénard, Caroline Giraud, Cédric Muller, Patrick Plésiat, Paulo Juarez

**Affiliations:** ^1^Département Recherche et Développement, Smaltis SAS, Besançon, France; ^2^Laboratoire de Bactériologie, UMR CNRS 6249 Chrono-Environnement, Université Bourgogne Franche-Comté, Besançon, France; ^3^Normandie Université, UNICAEN, U2RM, Caen, France; ^4^Centre National de Référence de la Résistance aux Antibiotiques, Centre Hospitalier Régional Universitaire de Besançon, Besançon, France

**Keywords:** *Pseudomonas aeruginosa*, genome reduction, virulence, antibiotic resistance, biotechnology

## Abstract

*Pseudomonas aeruginosa* is a human opportunistic pathogen responsible for nosocomial infections, which is largely used as a model organism to study antibiotic resistance and pathogenesis. As other species of the genus, its wide metabolic versatility appears to be attractive to study biotechnological applications. However, its natural resistance to antibiotics and its capacity to produce a wide range of virulence factors argue against its biotechnological potential. By reducing the genome of the reference strain PAO1, we explored the development of four hypovirulent and hypersusceptible recombinant DNA hosts (rDNA hosts). Despite deleting up to 0.8% of the core genome, any of the developed strains presented alterations of fitness when cultured under standard laboratory conditions. Other features such as antibiotic susceptibility, cytotoxicity, *in vivo* pathogenesis, and expression of heterologous peptides were also explored to highlight the potential applications of these models. This work stands as the first stage of the development of a safe-platform strain of *Pseudomonas aeruginosa* that will be further optimized for biotechnological applications.

## Introduction

Since the pioneering works of Bruce Holloway in the 1950s, *Pseudomonas aeruginosa* has become a model organism for exploring the genetics and physiological functions of genus *Pseudomonas*, and more generally Gram-negative non-fermenters. However, this microorganism stands out from most of related species by its capacity to adapt to and thrive in multiple environments, including the hospital (Moradali et al., 2017). This last adaptation makes of this bacterium an opportunistic pathogen able to cause life-threatening infections in immunocompromised patients such as those with respiratory infections, bacteremia and chronic diseases such as cystic fibrosis (Lister et al., 2009; de Bentzmann and Plésiat, 2011; McCarthy, 2015). Despite the development of specific molecular biology tools, the exploitation of the metabolic versatility of *P. aeruginosa* to produce recombinant proteins under safe conditions has been hampered by the potential pathogenicity of the bacterium (Moradali et al., 2017). The remarkable adaptability of *P. aeruginosa* is determined by a large genome of 6.4- to 7.4-Mbp in size (Hilker et al., 2015), the annotation of which is still in progress^1^ (Stover et al., 2000). It harbors genes encoding for a wide range of secreted virulence factors including lipases, proteases, exoenzymes and exotoxins (Dacheux et al., 2001; Diggle et al., 2006; Chemani et al., 2009; Hauser, 2009; Filloux, 2011; Burrows, 2012). Moreover, the cell envelope of this bacterium also contains several virulence factors such as adhesins, lectins, pili, flagella, and lipopolysaccharide (LPS) in the outer membrane (Feldman et al., 1998; Kohler et al., 2000; King et al., 2009; Wilder et al., 2011; Burrows, 2012; Kazmierczak et al., 2015). Additionally, to its pathogenesis, *P. aeruginosa* possesses an intrinsic resistance to several anti-Gram-negative antibiotics such as β-lactams and aminoglycosides (Lister et al., 2009; de Bentzmann and Plésiat, 2011; Li et al., 2015; McCarthy, 2015). This natural resistance is principally due to the low permeability of its membrane, the production of enzymes capable to degrade or modify antibiotics and the action of two efflux systems able to export multiple molecules outside the cell (Yonezawa et al., 1995; Poole, 2004; Juan et al., 2005, 2006; Lister et al., 2009; Vettoretti et al., 2009; Berrazeg et al., 2015; Ropy et al., 2015; Jeannot et al., 2017). All these genomic determinants were targeted in this work with the objective to build up four different rDNA hosts by reducing the genome of the reference strain PAO1 without causing any negative impact on bacterial growth very similar as the work done on *P. putida* (Lieder et al., 2015). This project is placed as a first stage to develop a safe-platform strain of *Pseudomonas aeruginosa* which will be further optimized for biotechnological applications such as expression of heterologous proteins or secondary metabolites.

## Materials and Methods

### Bacterial Strains, Plasmids, and Growth Conditions

Main features of bacterial strains and plasmids used in this study are listed in Table 1. Bacterial cultures of *E. coli* were performed in Luria-Bertani broth or on Luria-Bertani agar while those of *P. aeruginosa* were performed in Mueller-Hinton broth (MHB) with adjusted concentrations of Ca^2+^ (from 20 to 25 μg mL^–1^) and Mg^2+^ (from 10 to 12.5 μg mL^–1^), on Mueller-Hinton agar (MHA) or on Typticase Soy Broth (TSB). All media were purchased from Beckton-Dickinson. When needed, 50 μg mL^–1^ kanamycin or 50 μg mL^–1^ streptomycin was added to *E. coli* growth media. Recombinant plasmids were introduced into *P. aeruginosa* by triparental mating and mobilization with conjugative plasmid pRK2013 (Km^*R*^) provided by helper strain *E. coli* HB101 (Ditta et al., 1980) or electroporation (Dennis and Sokol, 1995).

**TABLE 1:** Strains and plasmids used in this study.

Strains	Relevant characteristics	Sources or references
**Pseudomonas aeruginosa**		
PAO1	Wild-type reference strain, prototroph	Stover et al., 2000
SMEff	PAO1Δ*mexAB,mexCD*,*mexEF,mexXY*	This study
SMRes	SMEffΔ*ampC*,*aph,arnBCADTEFugd*	This study
SMVir	PAO1Δ*exoS,exoT,exoY,fliEFG,lasA*,*pilQ,plcH*,*pqsA*,*rhlA,toxA*	This study
SM54	SMRes-VirΔ*lecA*,*lecB,pcrVHpopBD*	This study
***Escherichia coli***		
DH5α	F^–^ *ϕ*80*lac*Z*Δ*M15 Δ(*lacZYA*-*argF*)U169 *rec*A1 *end*A1 *hsd*R17(r_*k*_^–^, m_*k*_^+^) *pho*A *sup*E44 *thi*-1 *gyr*A96 *rel*A1 λ^–^	Invitrogen
CC118	Δ(*ara-leu*) *araD* Δ*lacX74 galE galK phoA20 thi-1 rpsE rpoB argE* (Am) *recA1*	Manoil and Beckwith, 1985
CC118λpir	Δ(*ara-leu*) *araD* Δ*lacX74 galE galK phoA20 thi-1 rpsE rpoB argE* (Am) *recA1*, lysogenic for phage λpir	Herrero et al., 1990
HB101	*supE44 hsdS20*(r_*B*_^–^ m_*B*_^–^) *recA13 ara-14 pro A2 lacY1 galK2 rps*L20 *xyl-5 mtl-1 leuμB6 thi-1*	Lacks and Greenberg, 1977
**Plasmids**		
pRK2013	Helper plasmid for mobilization of non-self-transmissible plasmids, *mob1, tra1 col E1*, Km^*R*^	Kaniga et al., 1991
pKNG101	Suicide vector *oriR6K sacB insB*, Sm^*R*^	Kaniga et al., 1991

*Km^*R*^: kanamycin resistance; Sm^*R*^: streptomycin resistance.*

### Genomic Deletions

**FIGURE 1:**
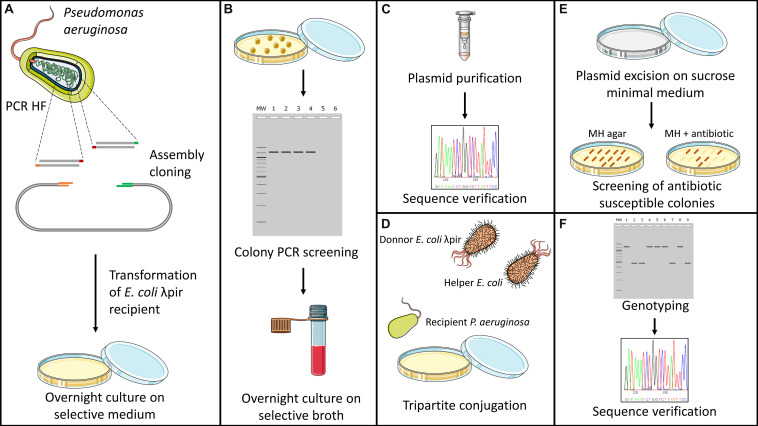
Schematic representation of genome deletion protocol. Genome deletions were carried out by homologous recombination using plasmid pKNG101 as a suicide vector (Kaniga et al., 1991). **(A)** Firstly, upstream and downstream regions of the targeted gene are amplified using a high-fidelity polymerase. Purified fragments are then inserted into linearized plasmid by assembly cloning and used to transformed CC118λpir competent cells of *E. coli*. **(B)** Antibiotic resistant colonies obtained after transformation are then screened by colony PCR to confirm the insertion of both fragments into the vector. **(C)** Recombinant plasmids are extracted and verified for absence of undesired SNPs through Sanger sequencing. **(D)** Transfer of recombinant plasmid to *Pseudomonas aeruginosa* is made by tripartite conjugation using the donor CC118 λpir strain of *E. coli* and HB101 helper strain containing plasmid pRK2013. **(E)** Transconjugants are then placed on M9 Minimal Medium containing sucrose as sole source of carbon. After several days of culture at room temperature, colonies are screened for plasmid excision through antibiotic susceptibility testing on nutritive media. **(F)** Antibiotic-susceptible colonies are screened through colony PCR for deletion of the targeted gene. Chromosomic deletions are verified by Sanger sequencing.

Gene inactivation experiments were all carried out using the suicide plasmid pKNG101 (Kaniga et al., 1991) and homologous recombination events, Figure 1 schematizes all the steps involved in the gene deletion protocol. Additionally, the map of plasmid pKNG101 is presented in Supplementary Figure 1. Briefly, recombinant plasmids were constructed by assembly cloning using the NEBuilder Hi-Fi DNA Assembly Cloning Kit (New England Biolabs). Assembly products were directly used to transform competent *E. coli* CC118λ*pir* strains. Once checked by DNA sequencing, recombinant plasmids with appropriate inserts were transferred to *P. aeruginosa* by conjugation. Transconjugants were selected on *Pseudomonas* Isolation Agar (PIA; Becton Dickinson) containing 500 or 2,000 μg mL^–1^ streptomycin. Excision of the undesired pKNG101 sequence was obtained by plating transformants on M9 plates (8.54 mM NaCl, 25.18 mM NaH_2_PO_4_, 18.68 mM NH_4_Cl, 22 mM KH_2_PO_4_, 2 mM MgSO_4_, 0.8% agar, pH 7.4) containing 5% (wt/vol) sucrose. Negative selection on streptomycin-containing MHA allowed the identification of transconjugants that had lost the plasmid. Finally, the allelic exchanges were verified by PCR and sequencing. All genomic modifications made to strain SM54 were verified by Whole Genome Sequencing using Illumina NextSeq sequencing (reads of 150 bp and 100× coverage). Reads were mapped against the PAO1 genome (Refseq NZ_CP053028.1) using CLC Genomic Workbench. Supplementary Table 1 lists the primers and temperatures employed to amplify the DNA fragments that were cloned in pKNG101, and that flanked targeted genes in the PAO1 genome. Detailed information about genomic deletions is found on Supplementary Table Gene Deletion.

### Antibiotic Susceptibility Testing

The minimal inhibitory concentrations (MICs) of selected antibiotics were determined by the standard serial twofold dilution method in MHA with inocula of 10^4^ CFU per spot, according to the CLSI guidelines (Clinical and Laboratory Standards Institute, 2015). The presence of colonies was visually assessed after 18 h of incubation at 35°C.

### Virulence Assays

*Motility tests*. Swarming and swimming motilities of PAO1 and SM mutants were assessed as previously described (Kohler et al., 2000). Evaluation of twitching motility was performed by vertically inoculating the center of a Luria-Bertani agar plate (1% agar) down to the plastic with a colony collected on a toothpick (Déziel et al., 2001). After 24 h of growth at 37°C, the bacteria adherent to polystyrene were stained with a 1% (wt/vol) solution of crystal violet. Motility assays were all repeated twice. *Rhamnolipid production*. Production of rhamnolipids was detected by using a cetyltrimethylammonium (CTAB) agar medium (Kohler et al., 2000). Diffusion of rhamnolipids from bacterial spots after 24 h incubation at 37°C generated blue halos in the medium. *Hemolytic activity*. The capacity of strains to lyse red blood cells was assessed by streaking bacteria on Columbia Blood Agar plates. The test was considered positive when a clear halo was apparent around the colonies after an incubation of 18 h at 37°C.

### Determination of Bacterial Growth

*Growth curves in different culture media*. Bacteria were grown in MHB or TSB, respectively at 30^*o*^C and 37^*o*^C with shaking (225 rpm). Bacterial density was recorded at DO_600__*nm*_ in covered PS 96-well microplates (Nest), using a Spark 10M microplate reader (Tecan). *Biomass determination*. Strains were grown aerobically in 50 mL of TSB during 24 h, then pelleted at 11,000 × *g* for 10 min, and dried at 85°C until the pellet weight remained constant over 72 h. Presented results are means of two independent experiments and are expressed in grams of dehydrated bacteria per L of culture. *Bacterial counting*. Strains were aerobically cultured in MHB or TSB for 24 h at 30°C or 37°C. Colony forming units were then enumerated on agar plates in quadruplicates, by serial dilutions. Presented results are means of three independent experiments ± standard deviations.

### Transmission Electron Microscopy

Bacterial overnight cultures were adjusted at DO_600__*nm*_ = 1 in MHB. Bacteria were recovered by centrifugation prior to fixation 2.5% glutaraldehyde for 1 h at room temperature. Negative staining was done with a 1% aqueous solution of ammonium molybdate. Subsequently, the length of bacteria was measured in 100 randomly selected cells in each sample using a Hitachi H7800 at 80 kV equipped with LaB6 electron source.

### Recombinant Protein Production by Arabinose

The gene encoding the mCherry fluorescent protein was cloned on the arabinose-inducible expression vector pJN105 (Newman and Fuqua, 1999). The recombinant plasmid was next introduced into strains PAO1 and SM54 by electroporation. Log phase bacteria were resuspended at a cell density of OD_600__*nm*_ = 0.1 in MHB supplemented with 10 μg mL^–1^ of gentamicin for PAO1 or 2 μg mL^–1^ for SM54, and incubated with shaking (225 rpm) for 8 h at 37°C. The fluorescence of induced (0.5% wt/vol arabinose) and non-induced cultures was recorded with a Tecan Spark 10M microplate reader (excitation *A*_590__*nm*_/emission *A*_610__*nm*_). In parallel, bacterial growth was spectrophotometrically monitored at OD_600__*nm*_.

### Cytotoxic Activity on J774 A.1 Macrophages

Cell viability of J774 A.1 murine macrophages after bacterial infection was determined by using the Cytotoxicity Detection Kit (Sigma Aldrich), following suppliers’ recommendations. Briefly, 4 × 10^4^ cells were cultivated in RPMI 1640 medium (Gibco) supplemented with 1% of fetal bovine serum (FBS, Gibco) at 37°C for 24 h in a humid atmosphere (95%) enriched with 5% CO_2_. After incubation, macrophages were infected during 3 h with a Multiplicity of Infection of 5 (MOI = 5). The cytotoxicity of each bacterial strain was reported as a percentage of the total cell lysis caused by a Triton X-100 treatment. Tests were performed three times with two replicates.

### Adhesion on A549 Human Pulmonary Cells

Adhesion of bacteria was evaluated on human pulmonary cell lines A549. Briefly, 2.5 × 10^4^ cells were cultured in DMEM F-12 medium (Gibco) supplemented with 2 mM GlutaMAX (Gibco) and 10% FBS, at 37^*o*^C for 48 h in a humid atmosphere (95%) containing 5% CO_2_. Once at confluence, cells were infected during 2 h at MOI = 10. Supernatants containing non-adherent bacteria were collected, and cells were washed three times with 100 μL PBS (Gibco). Cell monolayers with adherent bacteria were then lysed using Triton X-100 0.1%. Bacteria of the collected aliquots were counted on MH plates by serial dilution. Bacterial adherence was reported as the percentage of CFU present in cell lysates, compared to the total of CFU counted in aliquots (lysate plus supernatants).

### *Galleria mellonella* Infection Assay

In-house reared *Galleria mellonella* larvae were infected subcutaneously using a syringe pump (KD scientific) with strains PAO1 and SM54, respectively. Bacteria were collected from overnight cultures by centrifugation, then washed and resuspended in physiological water in order to inoculate 30 CFU into each larva. Twenty insects were used per strain, and the experiments were repeated four times. Larvae killing was then monitored between 19 and 24 h post-infection. Data and statistical analysis were performed using the Kaplan-Meier R package and the logrank test, respectively.

### *In vivo* Evaluation of Pathogenesis in Murine Model

A total of 20 mice CD1 (female 28–32 g) were used to evaluate pathogenesis of strains PAO1 and SM54 of *P. aeruginosa*. Inocula were prepared by adjusting a bacterial suspension to OD_595__*nm*_ = 0.45 from an overnight solid culture. An additional 1:10 serial dilution was performed using cold physiological water to obtain a solution equivalent to 5 × 10^7^ CFU/mL. Each individual received 500 μL intraperitoneal injections equivalent to 2.5 × 10^7^ CFU. After inoculation, animals were monitored for mortality every 12 h for 4 days. The animal study was reviewed and approved by the Comité D’Ethique Régional (C2EA) and the Ministère de l’Enseignement Supérieur et de la Recherche (ref APAFIS#27692-2020101510342409 v2). Statistical analysis was performed using the logrank test.

## Results

### Targeted Gene Reduction Strategy

**FIGURE 2:**
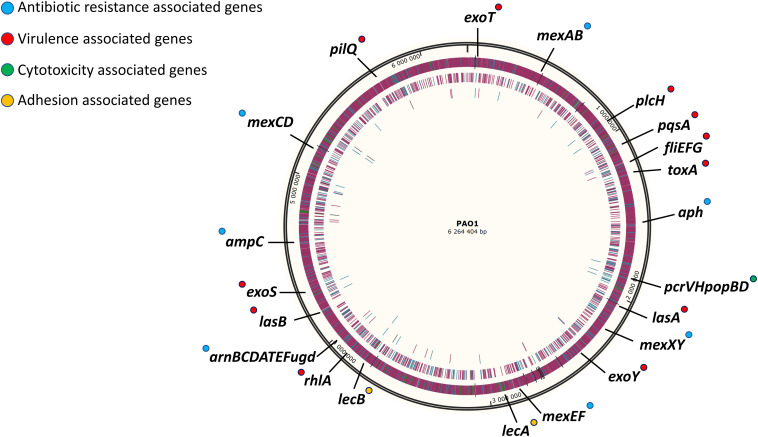
Schematic representation of targeted genomic regions for gene inactivation in strain SM54. Approximative position of the 37 genes deleted from the wild-type strain PAO1 in the physical map of its chromosome (Accession No. NC_002516). Genes related to antibiotic resistance are marked in blue, those related to virulence factors are marked in red, those related to cytotoxicity are marked in green and finally those genes related to adhesion are marked in yellow.

The strategy used in this work for gene deletion was the classical genome editing tool by homologous recombination, using a suicide plasmid with R6K origin and the counter-selection gene *sacB* responsible for saccharose susceptibility in various Gram-negative bacteria (Kaniga et al., 1991). The main molecular mechanisms driving antibiotic resistance and virulence factor production in *P. aeruginosa* have been largely studied and characterized (Lister et al., 2009; McCarthy, 2015). However, as *P. aeruginosa* possesses one of the most complex regulation network (Schuster and Greenberg, 2006; Huang et al., 2019; Kostylev et al., 2019) and some regulation pathways await to be described, targeting regulatory genes was not favored because of the probability to have non-desired effects. Instead, the main effector genes conducing to these traits of pathogenicity were removed in different blocks. First, two blocks of antibiotic resistance-associated genes were targeted; the four clinically significant RND efflux pumps were deleted in strain SMEff, namely MexAB, MexCD, MexEF and MexXY while other resistance genes concerning enzymatic (*ampC, aph*) and membrane-modification (*arnBCDATEFugd*) mechanisms were additionally removed in strain SMRes. A third block of gene deletions was performed to obtain strain SMVir in which virulence-associated genes were removed such as those involved in mobility and attachment (*fliEFG, pilQ*), secreted toxins (*plcH, exoT, exoS, exoY, toxA*), the *Pseudomonas* quinolone system (*pqsA*) and production of rhamnolipids (*rhlA*) and elastase (*lasA, lasB*). Finally, in addition to the above mentioned genes, a fourth block was targeted in strain SM54 and consisted in cytotoxicity related genes (*pcrVHpopBD*) and adhesion related genes (*lecA, lecB*). In SM54, a total of 37 genes were deleted (≈51 kb) which represents 0.8% of the core genome of the species. A scheme identifying all the effector genes removed in SM54 is shown in Figure 2.

### Reduction of Basal Antibiotic Resistance and Virulence Factor Production

As several effector genes were targeted for deletion in the different strains, determination of minimal inhibitory concentrations (MIC) and specific *in vitro* virulence assays were performed to verify the impairment of antibiotic resistance and production of virulence factors, respectively. As shown in Table 2, the efflux deficient strain SMEff was, from 2- to 32-fold, more susceptible to all antibiotics tested, these included antibiotics commonly used as resistance markers in molecular biology (ampicillin, chloramphenicol, tetracycline, streptomycin, and kanamycin) and clinically used antipseudomonals (gentamicin, tobramycin, ceftazidime, meropenem, aztreonam and colistin). It was interesting to notice that even though several other genes were deleted from SMRes and SM54 strains, only their susceptibility to ampicillin and kanamycin was highly increased (more than 1000-fold and 64-fold, respectively) in comparison to SMEff. Concerning strain SMVir, its susceptibility to antibiotics remained unchanged as expected. The production of several virulence factors was also assessed by performing different *in vitro* tests. As it was intended, different types of motilities such as swarming, swimming, and twitching dependent on flagellum and pili were abolished in strains SMVir and SM54. The same was observed for the production of hemolytic factors and rhamnolipids (Supplementary Figure 2). Not surprisingly, none of these features were altered in strains SMEff and SMRes (data not shown).

**TABLE 2:** Antibiotic susceptibility of selected strains.

Strain	Minimal inhibitory concentration (mg L^–1^)*										
	Commonly used in molecular biology					Commonly used as antipseudomonal					
	AMP	CHL	TET	STR	KAN	GEN	TMN	CAZ	MEM	ATM	CST
PAO1	4,096	32	32	32	128	1	0.5	1	0.5	4	1
SMEff	1,024	2	1	4	64	0.25	0.125	0.5	0.03	0.25	1
SMRes	0.25	2	1	4	1	0.25	0.125	0.5	0.03	0.25	1
SMVir	4,096	32	32	32	128	1	0.5	1	0.5	4	1
SM54	0.25	2	1	4	1	0.25	0.125	0.5	0.03	0.25	1

*Values from three independent experiments. AMP, ampicillin; CHL, chloramphenicol; TET, tetracycline; STR, streptomycin; KAN, kanamycin; GEN, gentamicin; TMN, tobramycin; CAZ, ceftazidime; MEM, meropenem; ATM, aztreonam; CST, colistin.

### Effect of Genome Reduction on Biomass and Bacterial Morphology

**FIGURE 3:**
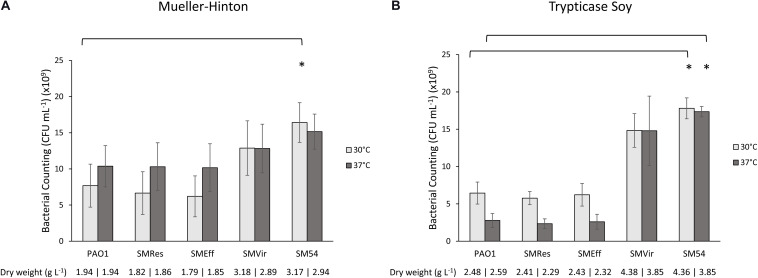
Bacterial counting and dry weight at 24 h of growth. Bacterial cells were counted after 24 h of culture at 30°C (light gray) or at 37°C (dark gray) in **(A)** Mueller-Hinton Broth and **(B)** Trypticase Soy Broth. All cultures were agitated at 225 rpm to allow proper oxygenation. Results represent mean values of three independent experiments. Error bars represent standard deviation and asterisks (*) represent a significant difference in the number of CFU counted between the two strains compared according to a Student’s *t* test (*p* < 0.01). Below each graph, dry weights produced by these cultures are indicated.

As genome reduction may provoke deleterious effects for biotechnological applications such as growth, biomass production and cellular morphology (Kurokawa et al., 2016), some of these parameters were evaluated. Bacterial counting and dry weight determination were thus performed for all the PAO1-derived strains. As *P. aeruginosa* is a non-fastidious bacterium to growth, two nutritive broths were evaluated: Mueller-Hinton (MHB) and Trypticase Soy (TSB) at two temperatures, 30°C and 37°C. Interestingly, as shown in Figure 3, after 24 h of incubation strains SMVir and SM54 produced significantly more bacterial counting (from 1.3- to 6.1-fold) and more dry weight (from 1.5- to 1.8-fold) compared to the parental strain PAO1. An important remark is that the best performance for these strains was observed when cultured in TSB at 37°C. Concerning strains SMRes and SMEff, their behavior was very similar to that of the wild-type strain PAO1. Finally, cell morphology was studied by transmission electron microscopy to evaluate if genomic modifications could modify this trait. Even though, 0.8% of the genome was withdrawn in SM54, no significant changes were observed compared to PAO1 (*p*-value >0.05) (Supplementary Figure 3).

### Genome Reduction Does Not Impair Heterologous Protein Production in SM54

**FIGURE 4:**
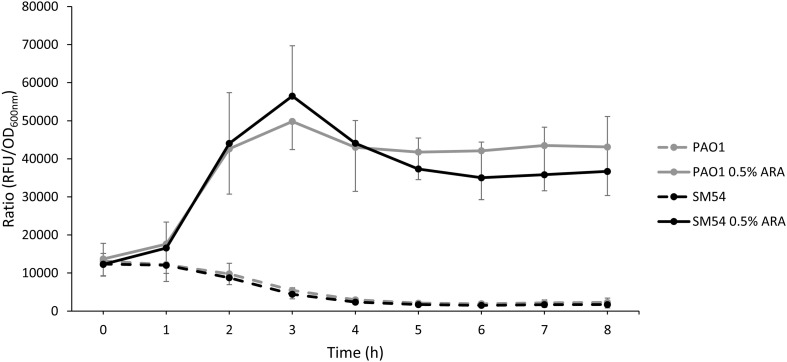
Accumulation of the red fluorescent protein mCherry. Strains PAO1 and SM54 were grown in Mueller-Hinton broth containing 10 or 2 μg mL^– 1^ of Gentamicin, respectively, used as selection marker of plasmid pJN105. The transcription of the mCherry coding gene was induced with 0.5% of arabinose and the accumulation of the fluorescent protein was monitored using a microplate reader (Ex 590 nm, Em 610 nm) (solid lines). Non-induced cultures were used as controls (dashed lines). Results represent mean values of three independent experiments and error bars represent standard deviation.

As strain SM54 showed interesting characteristics in all the aspects studied above, a step forward was taken with this strain to evaluate other features. The natural capacity of the modified strain to produce heterologous proteins was evaluated as this feature might be also affected by genome reduction (Lieder et al., 2015; Zhu et al., 2017). For this, the encoding sequence of the red fluorescent protein mCherry was inserted into the arabinose-inducing plasmid pJN105 and fluorescence was followed during 8 h. As shown in Figure 4 the ratio fluorescence/DO_600_ emitted by SM54 harboring this recombinant plasmid was very similar to that produced by the parental strain PAO1, thus providing evidence that the smaller genome of SM54 does not significantly impair protein synthesis.

### *In vitro* and *in vivo* Evaluation of SM54 Pathogenicity

**FIGURE 5:**
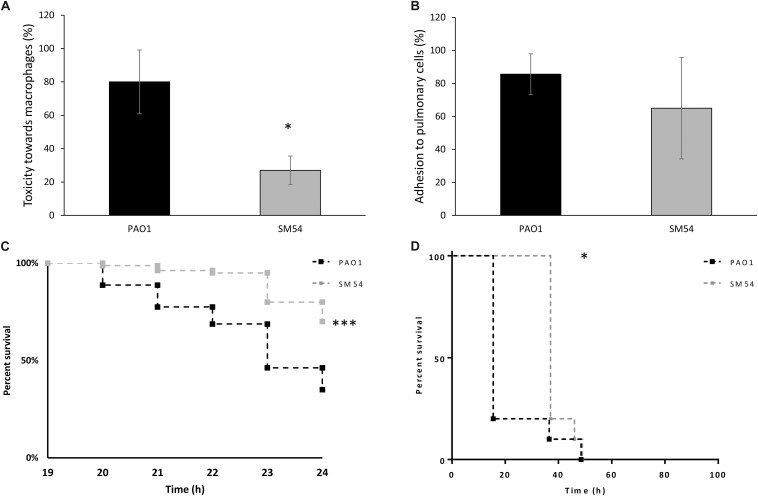
Evaluation of SM54 pathogenesis. **(A)** cytotoxicity tests performed on J774 A.1 murine macrophages using a MOI of 5. Results represent mean values of three independent experiments for which a significant difference was shown according to the Mann-Whitney test (*p* < 0.01) (*). **(B)** Adhesion tests performed on A549 human pulmonary cells using a MOI of 10. Results represent mean values of three independent experiments. Error bars represent standard deviation. **(C)** Kaplan-Meier survival curves of *G. mellonella* larvae infected with the WT strain PAO1 (black) or SM54 (gray). Results represent mean values from four independent experiments for which a significantly difference (***) was shown according to the logrank test (*p* < 0.001). **(D)** Kaplan-Meier survival curves of mice CD3 infected with the WT strain PAO1 (black) or SM54 (gray). Results represent the survival of 20 individuals for each condition. Survival between the two groups of organisms was shown to be significantly different after 15 h (*) of infection according to the logrank test (*p* < 0.05).

As SM54 showed potential for further biotechnological applications, it seemed to be essential to evaluate additional traits of pathogenicity. Thus, *in vitro* assays of cytotoxicity and cell adhesion were performed using murine macrophages (J774 A.1 cells) and human pulmonary cells (A549 cells), respectively. Once again, strain SM54 showed a reduction of these two pathogenic traits (Figure 5). A significant decrease of cytotoxicity was observed compared to PAO1 (27 vs 80%, respectively. *p*-value <0.05). On the other hand, concerning adhesion to pulmonary cells, even though a reduction of this trait was noted, the difference between these two strains was not statistically significant (PAO1 86% vs SM54 65%; *p*-value >0.05). Finally, to test whether the pathogenicity of SM54 was significantly attenuated, two *in vivo* models were studied. Firstly, larvae of *Galleria mellonella* were infected with 30 CFU and secondly CD1 mice were challenged with intraperitoneal injections of 2.5 × 10^7^ CFU of each strain; for both cases survival of individuals was followed over time (Figure 5). In the case of *G. mellonella* infection, it was observed that larvae infected with SM54 significantly survived 50% better than those infected with PAO1 after 24 h of infection (*p*-value <0.001). For CD1 mice infection, even though 100% of mortality was observed after 48 h of infection, the mortality of individuals infected with SM54 was significantly slower than those infected with PAO1 (0 vs 80% at 15 h, *p*-value <0.05).

## Discussion

Several biotechnological applications of the genus *Pseudomonas* have been largely studied, especially for *P. putida* (Poblete-Castro et al., 2012; Molina et al., 2013; Martinez-Garcia and de Lorenzo, 2019; Weimer et al., 2020). The species of this genus are highly adaptative to a wide-range of environments fact that could be explained by their large genome containing variable metabolic pathways which become active in response to the environmental conditions in which the bacterium is growing (Silby et al., 2011; Mielko et al., 2019). In the case of *P. aeruginosa*, the biotechnological applications have been poorly studied mainly because its character of opportunistic pathogen. In this study, we have sought to develop an attenuated strain derived from *P. aeruginosa* PAO1 as a first step to create a chassis for biotechnological applications. Genome reduction was the strategy used to target specific effector genes related to antibiotic resistance and pathogenicity. Thus, strain SM54 was engineered by sequentially deleting four blocks of genes related to these traits. Additionally, three other strains are also described, SMEff, SMRes and SMVir, which could be helpful to study isolated mechanisms of antibiotic resistance or virulence. Overall, the effects of these genomic deletions on antibiotic resistance and virulence showed the expected results. In detail, our results showed that when antibiotic related genes were targeted, as in SMEff, SMRes or SM54, the basal susceptibility to antibiotics was highly increased and, on the same logic, when virulence factor genes were deleted in strains SMVir or SM54 their capacities to produce these factors was abolished. It is important to notice that not all pathogenic traits were targeted in this first stage development. For example, another well-characterized determinant of antibiotic resistance of *P. aeruginosa* is gene *oprD* (PA0958) (Lister et al., 2009). It was here considered not remove *oprD* from SM54 as its deletion would result in a decrease of strain susceptibility to carbapenems. Indeed, this gene codes for the OprD substrate-specific porin which serves as the main portal of entry for basic amino acids and carbapenems (Fukuoka et al., 1993) and which when altered results in significant resistance to these antibiotics. Furthermore, *P. aeruginosa* also harbors in its genome 8 additional RND efflux systems (representing ∼ 44.8 kb) for which the contribution to antibiotic resistance and bacterial physiology has been poorly studied (Poole, 2014). For this reason, it was considered here that they should not be removed from SM54 to avoid undesirable effects on fitness or physiology. As previous studies have shown that genome reduction might have either positive or negative effects on bacterial development (Lieder et al., 2015; Kurokawa et al., 2016), we studied the effect of these modifications on biomass and bacterial morphology. Our results showed that when virulence related genes are deleted, the dry weight and the number of CFUs recovered after 24 h of culture are significantly increased when compared to strain PAO1. These results suggest that strains SMVir and SM54 may eventually produce larger amounts of biomass than a wild-type strain when cultured in rich media such as MH or TSB. It is important to notice that this yield could be modified following the nutrient level of the medium used (e.g., rich vs minimal media) as it was shown for genome reduced *E. coli* (Kurokawa et al., 2016; Tsuchiya et al., 2018) and further characterization of SM54 will explore this behavior. Concerning TEM observation of bacterial cells, even though the difference between PAO1 and SM54 was not statistically significant, SM54 cells are slightly bigger in length and width which is in accordance with other studies evaluating the impact of genome reduction on cell morphology (Hutchison et al., 2016; Kurokawa et al., 2016). Based on these observations, it would be expected that if additional deletions are made to SM54 genome, the morphology of the cells would have tendance to be bigger. Previous work done on *P. putida* showed that following genome reduction, the capacities of the strain to produce heterologous proteins were enhanced (Lieder et al., 2015). Following the same line of thinking, we evaluate the capacity of SM54 to produce the fluorescent protein mCherry after induction with arabinose. The difference between PAO1 and SM54 was not significant (*p*-value >0.05) suggesting that the genomic modifications done to SM54 did not have any effect on this trait. The deletion of non-essential genes was shown to improve the expression of heterologous genes in *P. putida* (Martinez-Garcia et al., 2014) it would be interesting to explore if such deletions would have the same positive effects on SM54. Bioinformatic analysis has shown that SM54 still harbors genes coding for two prophages (cluster 1 from PA0617 to PA0641 and cluster 2 from PA0720 to PA0728), two endonucleases (*endA, nth*), one restriction-modification system (*hdsMSR*) and about 7 kb coding for insertion sequences representing a total of ∼43 kb. Targeting these regions would be the next step to consider in the improvement of SM54 toward the application of recombinant protein production. Regarding pathogenesis experiments, our *in vitro* results showed that SM54 is no longer capable to induce cell death when tested against murine macrophages and is less adhesive to human pulmonary cells. We sought to further reduce the adhesion of SM54 by deleting genes related to biofilm formation (chaperone usher systems- strain SM59) (Vallet et al., 2001; Giraud and de Bentzmann, 2012). However, in SM59, bacterial growth was importantly impacted (data not shown) and deletion of the *cup* clusters provoked clumps in liquid cultures and roughly colonies clearly; showing that some deletions can be deleterious for bacterial fitness and further applications. Regarding these results, we kept SM54 to evaluate its pathogenesis *in vivo* using *Galleria mellonella* larvae and CD1 mice as models. The first has been mainly used to evaluate the response of innate immunity to bacterial infections (Tsai et al., 2016) and more specifically to study the role of the type III secretion system of *P. aeruginosa* pathogenesis (Miyata et al., 2003). The murine model has been now extensively used for study a wide variety of bacterial infections and allows the study of both innate and adaptative immune responses (Stotland et al., 2000). Although our results showed a decreased of mortality in both models, individuals infected with SM54 kept dying at a slower rate than those infected with PAO1. Recently, it has been showed that targeting five key pathogenicity genes from the *P. aeruginosa* chromosome was enough to produce an attenuated strain incapable to kill mice (Valentine et al., 2020). Among these genes, *phzM* encoding for a phenazine-specific methyltransferase required for pyocyanin production (Rada and Leto, 2013) and *wapR* encoding for a rhamnosyltransferase involved in the synthesis of the O antigen of LPS (Poon et al., 2008) could be two interesting targets to delete from SM54 to finally produce an attenuated strain. Overall, further genetic modifications of strain SM54 is undeniably needed to reduce its pathogenesis and to improve its capacities for heterologous gene expression. It should be noted that these modifications could have an impact on bacterial fitness and choices would be needed to equilibrate efficacy and yield of the desired product. The development of bacterial chassis requires a certain degree of engineering for which the actual state of SM54 might not be suitable to use this terminology. Indeed, as Synthetic Biology develops as science, the community tries to establish what a bacterial chassis is. Recently, it has been proposed that the term “chassis” should imply that the organisms labeled as such have undergone through several degrees of optimization from a genomic and metabolomic points of view. This kind of organisms should have a genome encoding a number of basic functions for stable self-maintenance, growth and optimal operation under specific environmental conditions (de Lorenzo et al., 2021). Following this definition, it is clear that SM54 is not and should not be called a chassis but rather a rDNA host for which one can use or develop specific plasmids to study heterologous gene expression, protein function or other fundamental studies. It is important to notice that by developing an attenuated strain, it is possible to make a pathogen like *P. aeruginosa* user-friendly and suitable for the use of classical tools of molecular biology (e.g., antibiotic cassettes). From a medical point of view, some studies have raised the possibility to use attenuated bacteria to deliver DNA plasmid vaccines (Dietrich et al., 2003). This approach has been already been explored for *P. aeruginosa* in which an attenuated strain deleted of gene *aroA*, involved in the synthesis of aromatic amino acids, was proven to be protective in a pre-clinical setting after challenging mice using models of corneal and acute lung infection (Priebe et al., 2002, 2003; Zaidi et al., 2006; Kamei et al., 2011). One can imagine that developing an unarmed chassis of *P. aeruginosa* such as SM54 could be a novel approach to further engineer this strain and explore this kind of medical applications. Even though the early stage of this project, SM54 proves to have some qualities which can be of interest for further optimization. As mentioned above, additional genomic modifications are planned (retirement of prophages, endonucleases, restriction-modification systems, insertion sequences, and abolishment of phenazine production and O antigen synthesis) to make of SM54 a safe-bacterial platform.

## Data Availability Statement

The original contributions presented in the study are included in the article/Supplementary Material, further inquiries can be directed to the corresponding author/s.

## Ethics Statement

The animal study was reviewed and approved by Comité D’Ethique Régional (C2EA) and the Ministère de l’Enseignement Supérieur et de la Recherche (ref APAFIS#27692-2020101510342409 v2).

## Author Contributions

MG, SG, and PJ performed the experimental procedures. CG performed *in vivo* survival tests on *Galleria mellonella*. CM, PP, and PJ designed the experiments. PJ wrote the manuscript. All the authors read the manuscript and agreed with its submission.

## Conflict of Interest

This work was done under industrial support from SMALTIS SAS; the distribution of biological material will be regulated by the establishment of Material Transfer Agreements after discussion with the company authorities.

## Footnotes

1 http://www.pseudomonas.com
