# Supplementary material for: Targeted Genome Reduction of Pseudomonas aeruginosa Strain PAO1 Led to the Development of Hypovirulent and Hypersusceptible rDNA Hosts
Source: Front Bioeng Biotechnol. 2021 Mar 11;9:640450. doi: 10.3389/fbioe.2021.640450 (PMC7991573; doi:10.3389/fbioe.2021.640450)
Supplement: Supplementary file 1 [file Table_1.DOCX]

Supplementary Material

**Table S1** Primers used in this study.

| **Name** | **Sequence (5’ 🡪 3’)** | **Tm (°C)** | **Sources** |
| --- | --- | --- | --- |
| **Inactivation** |  |  |  |
| PCRi MexXYC1 | CCAGCAACAGGTAGGGAGAA | 59 | This study |
| PCRi MexXYC2 | GCGGTACCAGGAATAGGGCGACCAG | 70 | This study |
| PCRi MexXYC3 | ATTCCTGGTACCGCTGTTCTTCCTGGT | 67 | This study |
| PCRi MexXYC4 | CAACGCCCGGAAGTTCTC | 59 | This study |
|  |  |  |  |
| INAC_mexAB_RD1 | GGCGTTTTTCATTGTGCTTC | 60 | This study |
| INAC_mexAB_RD2 | ACCGATCGTTGCATAGCGTTGTCCTCA | 72 | This study |
| INAC_mexAB_RD3 | ATGCAACGATCGGTACCGGCGTGAT | 72 | This study |
| INAC_mexAB_RD4 | GTAGCTGCGCTGGGTCAG | 60 | This study |
|  |  |  |  |
| INAC_mexCD_RD1 | GGAACATCTGCTCCAGGGTA | 58 | This study |
| INAC_mexCD_RD2 | CTCCCCGCACCGGCAACACCATTTC | 67 | This study |
| INAC_mexCD_RD3 | GCCGGTGCGGGGAGTGAACGAGATG | 72 | This study |
| INAC_mexCD_RD4 | ATCGTCGCTACCCAGCAG | 58 | This study |
|  |  |  |  |
| INAC_mexEF_RD1 | CTATTGATGCCGAACCTGCT | 58 | This study |
| INAC_mexEF_RD1 | AGAACACCCAGGAGAAGTGGGATGACT | 67 | This study |
| INAC_mexEF_RD1 | CTCCTGGGTGTTCTCCGGGATGATCG | 72 | This study |
| INAC_mexEF_RD1 | CGACTCCTTCTGGTTCTCCA | 58 | This study |
|  |  |  |  |
| INAC_ampC_RD1 | GAGGTGTCGAAGACGATGCT | 60 | This study |
| INAC_ampC_RD2 | TCACCCGAATCTGGTATCGCGCATGA | 72 | This study |
| INAC_ampC_RD3 | CCAGATTCGGGTGAAGATCGCCTAC | 72 | This study |
| INAC_ampC_RD4 | ATCCGCCTCGACGAACTG | 60 | This study |
|  |  |  |  |
| INAC_apH_RD1 | GATGGCCATGTGCTGACC | 58 | This study |
| INAC_apH_RD2 | CTAGAAGCATGGAGAAACGCTTCCTGT | 59 | This study |
| INAC_apH_RD3 | CTCCATGCTTCTAGAGTAGGGCCGGTTC | 59 | This study |
| INAC_apH_RD4 | GACTACGAAACGCTGCTCAA | 72 | This study |
|  |  |  |  |
| INAC_arn_A1 | AGATCATCGAGGTGCAGTCC | 58 | This study |
| INAC_arn_A2 | TGCGAGGGGAGAATGGCAGAAAGTCCA | 72 | This study |
| INAC_arn_A3 | ATTCTCCCCTCGCACAGGAAGATGG | 67 | This study |
| INAC_arn_A4 | TGTGCTTCATCTCCGAAGG | 57 | This study |
|  |  |  |  |
| INAC_lasA_RD1 | GAAACCCTGATCGACGAAAG | 58 | This study |
| INAC_lasA_RD2 | CAGTCGTCCAGCAAGACGAAGAGGAAC | 70 | This study |
| INAC_lasA_RD3 | TTGCTGGACGACTGTCGCCGCTACTAT | 71 | This study |
| INAC_lasA_RD4 | GTCGGAGTCCGGCTACTACG | 60 | This study |
|  |  |  |  |
| INAC_lasB_RD1 | CTCGACCAGTTGAACCAGGA | 59 | This study |
| INAC_lasB_RD2 | AAACACCCATGATCGCAACG | 58 | This study |
|  |  |  |  |
| INAC_fliEFG_RD1 | ACGTACGGGAACTCGACAAC | 58 | This study |
| INAC_fliEFG_RD2 | CTTCTGCTAGCAACAAGGGTGCCAAG | 68 | This study |
| INAC_fliEFG_RD3 | GTTGCTAGCAGAAGGAAATCCTCACCA | 67 | This study |
| INAC_fliEFG_RD4 | CTGGAGTCCATGTGCAGTTC | 58 | This study |
|  |  |  |  |
| INAC_pilQ_RD1 | GCCGATCTGCAGTCCTACAT | 58 | This study |
| INAC_pilQ_RD2 | TGATTATAGAGGCCACTGTTCATCGTC | 64 | This study |
| INAC_pilQ_RD3 | GGCCTCTATAATCAGGCCATCGCAATC | 69 | This study |
| INAC_pilQ_RD4 | TCGAGAATTTCCTGGACGAC | 58 | This study |
|  |  |  |  |
| INAC_pqsA_RD1 | TACGCAATGGGATTTCAACA | 58 | This study |
| INAC_pqsA_RD2 | CAACATGTGGCCCCGATAGTGATAAAC | 67 | This study |
| INAC_pqsA_RD3 | GGGGCCACATGTTGATTCAGGCTGTGG | 72 | This study |
| INAC_pqsA_RD4 | AGGTTGAGGTGTCCCTTGAC | 57 | This study |
|  |  |  |  |
| INAC_exoS_RD1 | AAACTAGTCTGTCAAGCAACTGCAAGG | 63 | This study |
| INAC_exoS_RD2 | AGTCCCTGGACTCTGCTGAAGCGATTG | 71 | This study |
| INAC_exoS_RD3 | AGAGTCCAGGGACTGCTCGATGCTCT | 69 | This study |
| INAC_exoS_RD4 | AAAGAGCTCTACCCTGCCGCTACTGAACT | 68 | This study |
|  |  |  |  |
| INAC_rhlA_RD1 | GCAGATGCTCTTCCTGCAAT | 59 | This study |
| INAC_rhlA_RD2 | CAAGGGTTGCAAACCGATACCAACAGA | 70 | This study |
| INAC_rhlA_RD3 | GTTTGCAACCCTTGACCTGCGAAGAC | 70 | This study |
| INAC_rhlA_RD4 | GTTGAACTTGGGGTGTACCG | 59 | This study |
|  |  |  |  |
| INAC_plcH_RD1 | CCTCGAAAGCGACTATCAGC | 59 | This study |
| INAC_plcH_RD2 | TTCAGGTCCAGTTTTCGGTCATCGTTT | 69 | This study |
| INAC_plcH_RD3 | AAACTGGACCTGAAGCGAGGAGTCCAT | 69 | This study |
| INAC_plcH_RD4 | GTATTCGCGCAGTTGCTCTT | 59 | This study |
|  |  |  |  |
| INAC_exoY_RD1 | ACATGGTCCACTTCCTCGTC | 59 | This study |
| INAC_exoY_RD2 | ACCTTACCGTTGCGTTAGAAACCACCT | 67 | This study |
| INAC_exoY_RD3 | CGCAACGGTAAGGTCTGATGCGCGAAG | 72 | This study |
| INAC_exoY_RD4 | GCAAGCCTTCGGAGTAAGTG | 58 | This study |
|  |  |  |  |
| INAC_exoT_RD1 | CAGCAAGGCTACCCAGACC | 59 | This study |
| INAC_exoT_RD2 | GATCCGTAAAGACGGGTTCTGCTGAGA | 69 | This study |
| INAC_exoT_RD3 | CGTCTTTACGGATCGTTCAGGCAAG | 67 | This study |
| INAC_exoT_RD4 | AGAGGGCGTAGTGAAAATCG | 58 | This study |
|  |  |  |  |
| INAC_exoS_RD1 | CTGTCCAGCAACTGCAAGG | 58 | This study |
| INAC_exoS_RD2 | AGTCCCTCAATCGCTTCAGCAGAGTCC | 66 | This study |
| INAC_exoS_RD3 | GCGATTGAGGGACTGCTCGATGCTCT | 67 | This study |
| INAC_exoS_RD4 | TACCCTGCCGCTACTGAACT | 60 | This study |
|  |  |  |  |
| INAC_toxA_RD1 | CCTCATCCTTCACCCATCAC | 58 | This study |
| INAC_toxA_RD2 | GTGATGGGTGAAGGATGAGG | 58 | This study |
|  |  |  |  |
| INAC_popBD_RD1 | TATCCTTCCCGCCTATCTGC | 58 | This study |
| INAC_popBD_RD2 | GTCACCTTCGGCGACATC | 58 | This study |
|  |  |  |  |
| INAC_lecA_RD1 | CCCTGCAGGTCGACGGCGACGCTGAGCAATAAGAAACC | 65 | This study |
| INAC_lecA_RD2 | TGCTAACAGGAAAGTGAAGTTGCCCGTGTG | 57 | This study |
| INAC_lecA_RD3 | CTTCACTTTCCTGTTAGCAAAGCACAGCAACG | 61 | This study |
| INAC_lecA_RD4 | TAATTCCACGGACTATAGACTATAATGAACCTGGTGATGAACGCG | 59 | This study |
|  |  |  |  |
| INAC_lecB_RD1 | CCCTGCAGGTCGACGGCTCGCCGATCCGCGCCTGGG | 72 | This study |
| INAC_lecB_RD2 | TCCGAACTCGGTGTATCTCCACTGAATACCTGGC | 61 | This study |
| INAC_lecB_RD3 | GATACACCGAGTTCGGAAGGGACGGGAT | 59 | This study |
| INAC_lecB_RD4 | TAATTCCACGGACTATAGACTATACTCTCGCAGATCATCGAACTGG | 59 | This study |


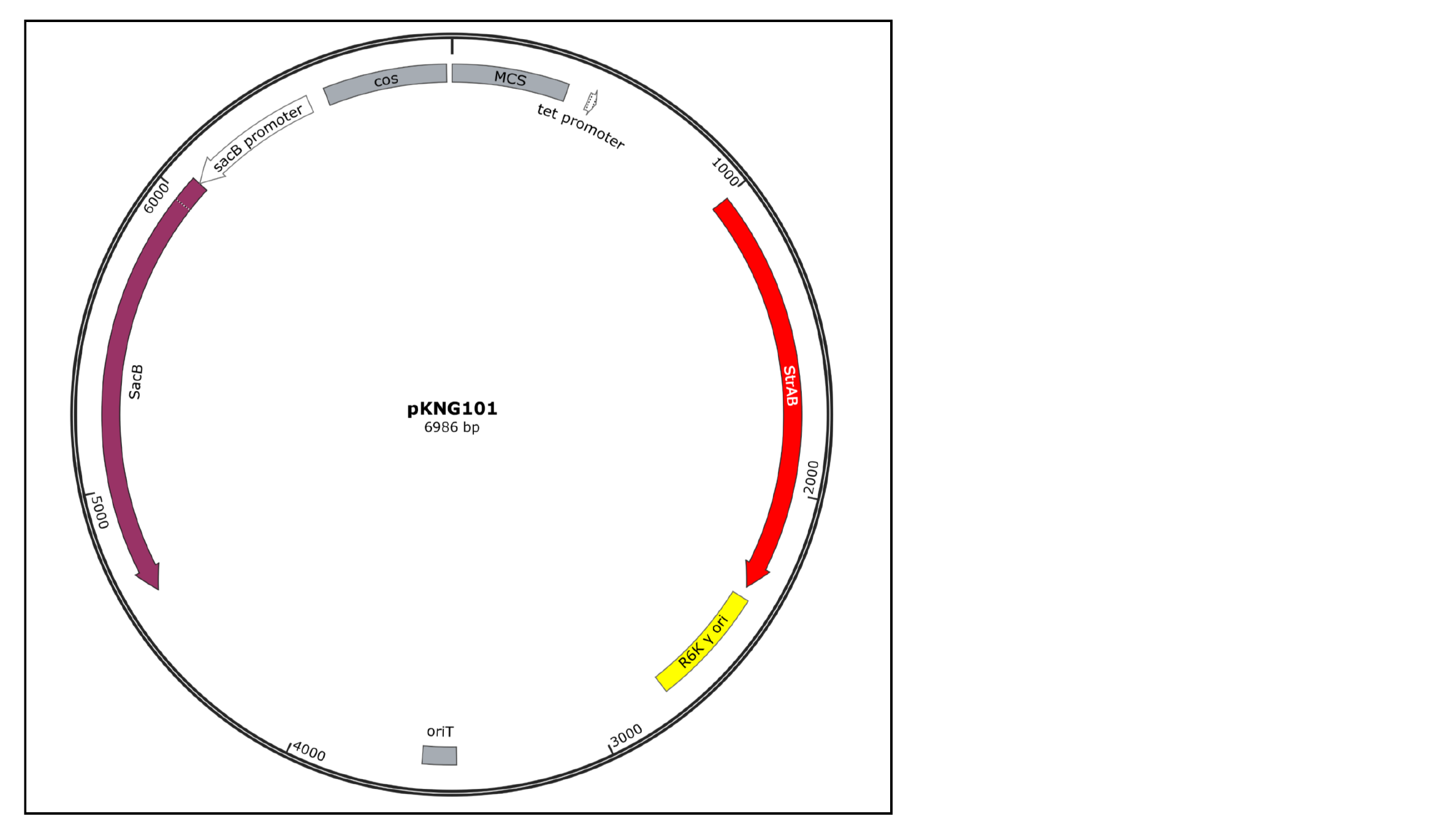


**Figure S1.** **Map of suicide plasmid pKNG101**. Suicide plasmid pKNG101 contains the R6K origin of replication which becomes functional only in the presence of protein π; thus, only strains harboring gene pir are able to replicate it (e.g., CC118λpir). Moreover, *oriT* allows this plasmid to be transferred to different recipients through bacterial conjugation. Gene *sacB* from *Bacillus subtilis* coding for levansucrase is used as counterselectable gene as it generates lethal sucrose sensibility in several Gram-negative species. Finally, genes StrAB are used as streptomycin resistance cassette.


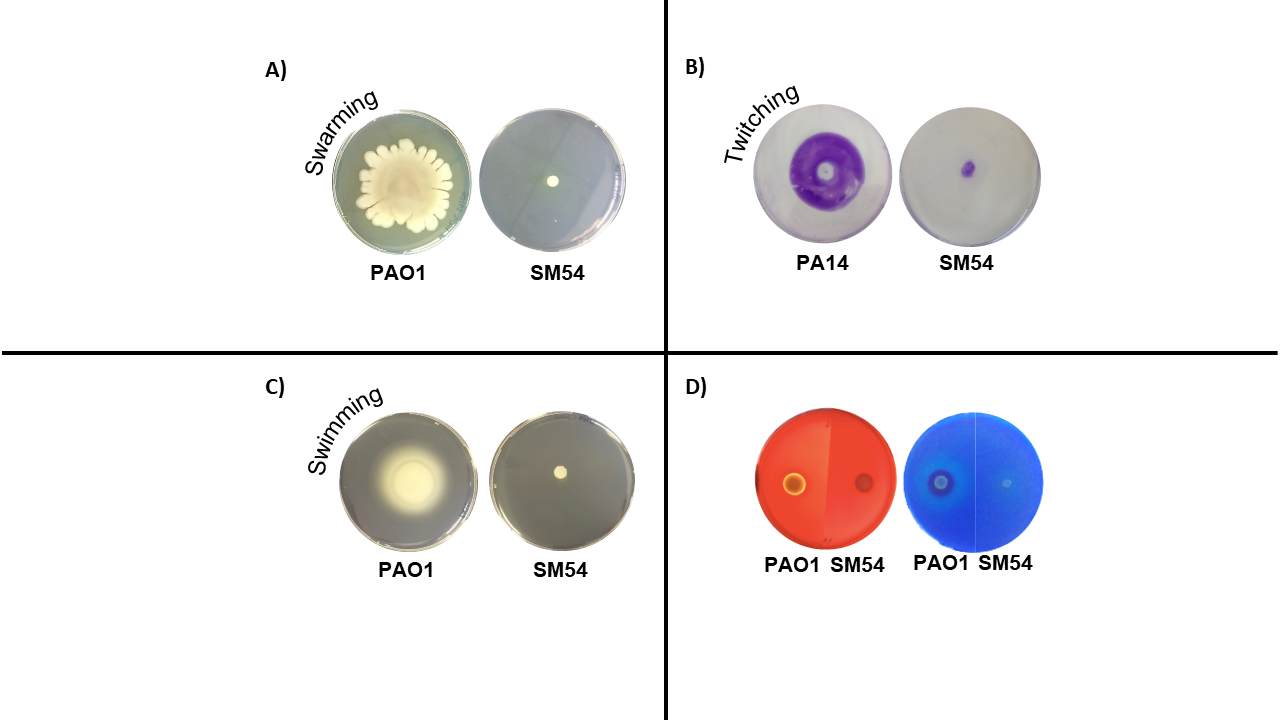


**Figure S2.** ***In vitro* evaluation of virulence**. Five traits of virulence were evaluated between WT strain PAO1 and the derived strain SM54. **(A)** Swarming motility, **(B)** Twitching motility, **(C)** Swimming motility and **(D)** Hemolytic activity (left, red plate) and production of rhamnolipids (right, blue plate).


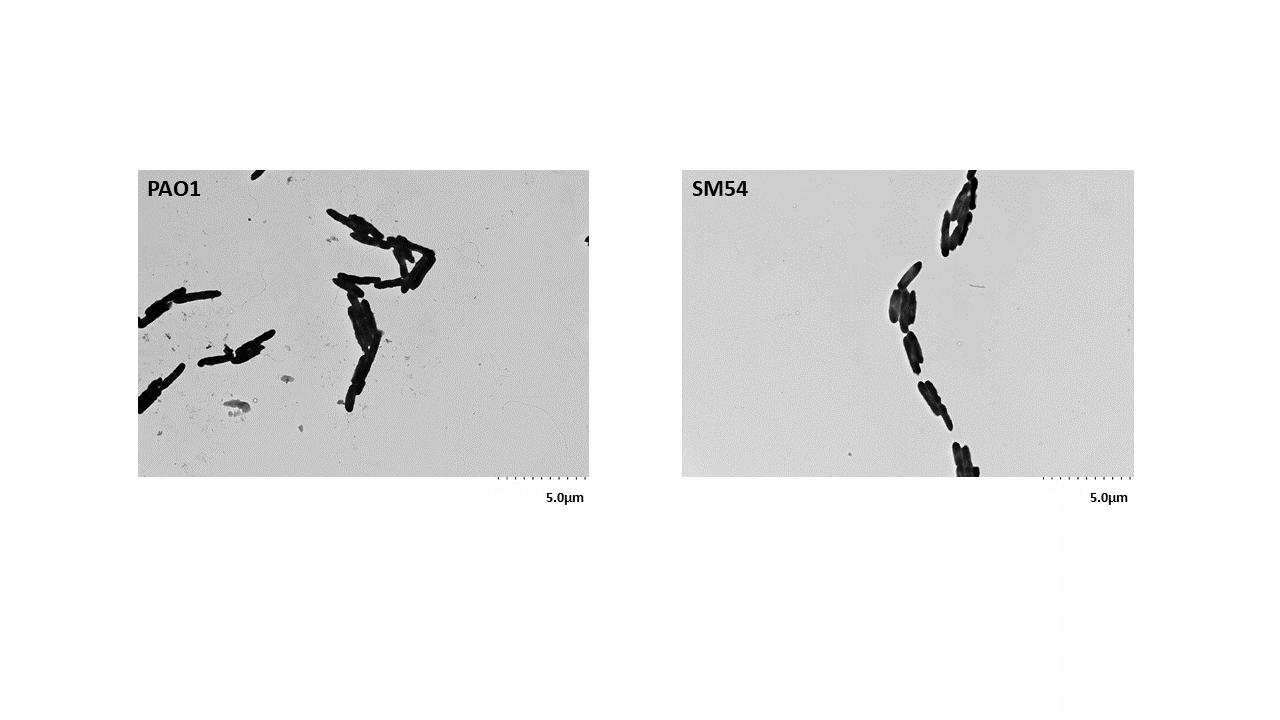


**Figure S3.** **Transmission electron microscopy of strains**. The morphology of strains PAO1 and SM54 was analyzed by transmission electron microscopy using negative staining. Measurements of length and width showed that there is no significant difference between these two bacterial cells (n=100, p-value > 0.05).
